# Effect of physical activity combined with extra ciliary-muscle training on visual acuity of children aged 10–11

**DOI:** 10.3389/fpubh.2022.949130

**Published:** 2022-08-30

**Authors:** Rongbin Yin, Jianrong Xu, Hongyun Wang, Sheng Zhou, Meng Zhang, Geng Cai

**Affiliations:** School of Physical Education, Soochow University, Suzhou, China

**Keywords:** children, physical activity, ciliary-muscle training, kinetic visual acuity, uncorrected distance visual acuity

## Abstract

This study is intended for exploring the effects of the physical activity combined with extra ciliary-muscle training with different frequencies on children's kinetic visual acuity and uncorrected distance visual acuity, and eventually figuring out the optimal frequency of ciliary-muscle training for each physical education class. To do the present research, A total of 160 students aged 10–11 from a school in Suzhou (a major city located in southeastern Jiangsu Province, East China) were randomly selected and divided into control group (*n* = 33), 15-frequency group (*n* = 44), 30-frequency group (*n* = 40) and 60-frequency group (*n* = 43), and the latter three experimental groups participated in a specially designed physical activity plan based on the training principles of ciliary muscle, while the control group participated in normal physical activity as usual. The experimental intervention period was 16 weeks, and all students' kinetic visual acuity and uncorrected distance visual acuity were measured before and after the experiment. The result showed that the kinetic visual acuity of the students in the 30 and 60-frequency groups got improved significantly after the experiment (*p* < 0.05), with the highest improvement occurring in the 30-frequency group, while there was no significant change in the 15-frequency group and the control group; The uncorrected distance visual acuity of the students in the 30 and 60-frequency groups was significantly improved after the experiment (*p* < 0.05), and the improvement range in these two groups was similar. In contrast, there was no significant change in the 15-frequency group, while the control group showed a significant decrease (*p* < 0.05). Physical activity combined with extra ciliary-muscle training has a positive effect on improving children's vision; at the same time, ciliary-muscle training with different frequencies bring out different outcomes on children's vision improvement, among which ciliary-muscle training with frequency of 30 in each physical education class is the best choice to enhance children's kinetic visual acuity and uncorrected distance visual acuity.

## Introduction

Eye health is an essential part of citizens' overall wellbeing, which has a far-reaching and extensive impact on individuals' life, health and society's sustainable development (1). In recent years, myopia has become a major public health issue worldwide. It is estimated that by 2050, the global myopia will increase significantly to 4.758 billion, accounting for 49.8% of the world's total population (2). Based on the investigations on current situation of myopia in the world, myopia presents clear regional characteristics, that is, the detection rate of myopia in Asia is much higher than that of Africa, Europe and America (3). For instance, in China, there are about 500 million patients suffer from refractive errors, of which 90% are myopic (4). Even worse, the morbidity of myopia is increasing, and persons with eye problems tend to be younger. The myopia rate of Chinese children and adolescents ranks first in the world (5). The large-scale prevalence of myopia has become a key factor triggering the downturn of eye health among children and adolescents.

With a progressive feature, the degree of myopia gradually rises with the increase of age, changing into moderate and high myopia, even into pathological myopia. The earlier children devolop myopia, the higher the risk of high myopia, pathological myopia and even blindness (6). Visual problems generate adverse effects on individual's health, wellbeing and economic development, including reduced educational and employment opportunities, social isolation and lower life expectancy, which will cause their quality of life to go down, and place a large barrier to the advancement of individuals, families and societies (7). In this situation, it is urgent to prevent the occurrence of children's myopia, slow down its progressive process and control the growth rate of myopia.

The vast majority of children's myopia is due to the long-term contraction of ciliary muscle under the condition of close visual activities without relaxing, which weakens the stretching ability of ciliary mustle and results in its loss of relaxation ability (8). In this case, improving the ciliary-muscle adjusting function is the key to prevent and slow down the formation of myopia (9). Kinetic visual acuity (KVA) and uncorrected distance visual acuity (UDVA), as the main indicators of visual acuity, are closely related to the adjusting function of ciliary muscle (10). Kinetic visual acuity is the ability to perceive the details of moving objects, which mainly depends on the regulating function of ciliary muscle (11), while the uncorrected distance visual acuity is the ability to perceive the details of static objects, which requires the ciliary-muscle adjustment to ensure a clear vision at both far and near visual distances. Kinetic visual acuity can help to predict uncorrected distance visual acuity to some extent (12). Existing studies have shown that the visual environment created in the process of physical activities is quite consistent with the principle of ciliary-muscle regulation, so specialized physical activities can improve people's kinetic visual acuity (13, 14), and also improve uncorrected distance visual acuity, as well as create a series of changes in eye parameters (15–19). Therefore, special physical activities designed under the guidance of ciliary-muscle regulation principle, can improve children's uncorrected distance visual acuity by promoting their kinetic visual acuity, which is an effective measure to ameliorate children's vision. Compared with ciliary-muscle training relying on apparatus (20, 21), physical exercise is more interesting, much safer, simpler and more acceptable to children, which can better benefit children and promote their visual health.

At present, the research on the prevention and control of children's myopia with physical activities mostly focused on impacts of different sports on children's visual acuity (22), but there are few quantitative studies on the intervention programs of physical activities. Obviously, to prevent and control children's myopia through physical activities, it is critical to define a reasonable range of intervention intensity, so as to make the myopia prevention and control program more practical and scientific. This study recorded and analyzed the effects of 15-frequency ciliary-muscle training, 30-frequency and 60-frequency ciliary-muscle training on children's kinetic visual acuity and uncorrected distance visual acuity in school physical education classes, and figured out the optimal threshold of training frequency of ciliary muscle in physical education classes, expecting to provide practical experience for the formulation of physical activities concerning the prevention and control of children's myopia.

## Materials and methods

### Participants

A total of 160 children aged 10–11 years old from four classes of Grade 5 at Experimental Primary School of Suzhou Science and Technology Town were randomly recruited as experimental subjects. Taking the class as a unit, they were randomly divided into three experimental groups and one control group (*n* = 33). The experimental included the 15-frequency group (low frequency, *n* = 44), the 30-frequency group (medium frequency, *n* = 40) and the 60-frequency group (high frequency, *n* = 43). Homogeneity tests were performed using one-way ANOVA. There was no significant (p>0.05)difference in the uncorrected distance visual acuity and kinetic visual acuity among the groups, which ensured that subsequent experiments could be carried out smoothly. Subjects' details are shown in Table 1.

**Table 1:** Participants demographic characteristics (*N* = 160, M ± SD).

**Variables**	**15-frequency group**	**30-frequency group**	**60-frequency group**	**Control group**	**Value of *P***
Total	44	40	43	33	
Boys	24	24	24	22	
Girls	20	16	19	11	
KVA	0.289 ± 0.246	0.268 ± 0.214	0.333 ± 0.304	0.274 ± 0.276	0.359
UDVA	4.875 ± 0.334	4.775 ± 0.306	4.807 ± 0.335	4.794 ± 0.302	0.766

KVA, kinetic visual acuity; UDVA, uncorrected distance visual acuity.

Inclusion criteria of eligible subjects in this study: (1) no astigmatism, amblyopia, hyperopia and other eye pathological symptoms; (2) no wearing orthokeratology lenses; (3) no cognitive and motor dysfunction, and being able to successfully complete the experimental tasks. This study protocol was in accordance with the *Declaration of Helsinki* and approved by the Ethics Committee of Soochow University (No.SU-DA20201010H01).

### Ciliary-muscle training method

Physical activity combined with ciliary-muscle training is designed in the present study, as studies have found that both closed-skill sports and open-skill sports can effectively improve students' kinetic visual acuity and uncorrected distance visual acuity after adding visual tasks (6). Generally, closed-skill sports include running, jumping and throwing. In detail, specific exercise tasks involve fast running, endurance running, obstacle running, standing long jump, single jump and double fall, rapid long jump, throwing sandbags or softball on the upper step, etc. On the strength of these regular physical activities, some visual targets were designed as digital cards, and some were printed action names, and solid balls, sandbags and other sports facilities were attached with visual targets signs as well. For example, in the obstacle running exercise, when students clearly saw the numbers and action names presented on the visual target in the process of running forward, which were set at each obstacle point in advance, and they should complete the described actions for the corresponding number of times. And the same to the jumping exercise and throwing exercise, students observed the number and content of the visual targets while jumping forward to complete the prescribed actions; during the throwing process, students were asked to track the movement of medicine balls, sandbags or softballs with eyes, carefully read the content of visual targets and did the written task on it.

Open-skill sports include football, basketball, volleyball and table tennis, the specific exercises of which are kicking the ball with the inside foot, catching the ball, dribbling the ball in front of the instep, dribbling the ball in basketball, shooting *in situ*, passing and catching the ball, self-cushion and two-person cushion in volleyball, throwing and catching ball and two-person play in table tennis. In these activities, the visual target was a card with numbers or a ball affixed with a visual-target design. Engaging in the exercise, students followed the movement of the ball with both eyes, or observed the hand-held visual target, and read the content of the visual target clearly (Table 2).

**Table 2:** Experiment content.

**Classification**	**Projects**	**Exercise content**	**Exercise time**	**Movement frequency**	**Duration of ciliary muscle intervention**
Open motor skills	Basketball	Passing, dribbling, shooting, etc.	Total 4 weeks; 40 min each time	Three times a week	Control group without ciliary muscle intervention and normal participation in physical education classes.
	Soccer	Shooting, passing, dribbling, etc.	Total 4 weeks; 40 min each time	Three times a week	The hourly value of ciliary muscle intervention in the experimental group was 3 s/time; since the ciliary muscle intervention was carried out as a motor exercise, the exact time could not be accurately estimated and depended on the content of the exercise. For example, the 60-frequency group performed volleyball reversal exercises with 40 people divided into four groups of 10 people at a time for 5 min each, for a total of 20 min.
	Volleyball	Matting, serving, passing, etc.	Total 4 weeks; 40 min each time	Three times a week	
	Table tennis	Throwing and catching balls, pairs, games, etc.	Total 4 weeks; 40 min each time	Three times a week	
Closed motor skills	Run	Obstacle run, speed run, relay run etc.	Total 4 weeks; 40 min each time	Three times a week	
	Jump	Height adjustment, long jump, etc.	Total 4 weeks; 40 min each time	Three times a week	
	Cast	Sandbag, softball, solid ball	Total 4 weeks; 40 min each time	Three times a week	
	Sports games	Emphasis on the far-sighted–near-sighted game	Total 4 weeks; 40 min each time	Three times a week	

The principles of the visual-task design and implementation consisted of four categories: (1) to ensure the completion of the exercise task and individual's safety; (2) regarding kinetic visual acuity, the change of depth or distance in the exercise was the main concern; (3) the emphasis of training placed on the changing process from “visible” to “see details of the visual target clearly”; (4) the times of each exercise was determined by the frequency of each group. Taking throwing and catching the ball as an example, the key of intervention is to guide the participant to track the movement and landing point of the ball with eyes when throwing and catching the ball. The ball from near to far or from far to near was recognized as one time of visual intervention.

### Study design

The determination of training frequency of ciliary muscle was in light of the practical experience of previous related studies, which indicated that the training frequency of ciliary muscle was generally 15–40 times in each physical education class according to different sports items and teaching contents (23, 24). Taking the limited time of physical education class and the requirements of teaching tasks into further consideration, this study would set the frequency of 30 as the benchmark threshold, and the upper and lower amplitudes were adjusted twice, so the experimental groups were assigned as the 15-frequency group (low frequency), the 30-frequency group (medium frequency) and the 60-frequency group (high frequency), respectively. In this situation, the effects of low, medium and high frequency of ciliary-muscle training on participants' kinetic visual acuity and uncorrected distance visual acuity were observed. The three experimental groups took part in additional ciliary-muscle training activities with the frequency of 15 (low frequency), the frequency of 30 (medium frequency) and the frequency of 60 (high frequency) designed based on the principle of ciliary-muscle training, while the control group carried out routine physical education activities.

For this study, the selected physical activities was from the regular school physical education for children aged 10–11, which consisted of open-skill sports like basketball, football, volleyball, table tennis and other ball games, and closed-skill sports, such as running, jumping, throwing in athletics. The intervention period of the experiment is 16 weeks, and the experimental group and the control group were arranged to conduct physical education class three times a week, with each class lasting 40 min. The intervention of the experimental group was carried out in physical education class. According to the research purpose, on the premise of not affecting the teaching content of normal physical education courses, combined with the characteristics of various sports movements and skills learning requirements, ciliary muscle training tasks were reasonably added to the experimental group in practice, and appropriate visual aids were added. Physical education class's exercises of the control group did not include ciliary muscle training tasks. For example, when the experimental group and the control group were practicing football passing at the same time, the football in the experimental group should be pasted with numbers or letters, and students were required to catch the moving track of the ball during the football rolling process. They need to see clearly the content of the visual mark on the football and experience the change of farsightedness and nearsightedness. However, there was no visual mark on the football in the control group, because the students in the control group did not need to complete the ciliary muscle training task. The exercise load, exercise duration, exercise frequency and items of the experimental group and the control group should be consistent to ensure the accuracy of the experimental results. At the same time, we keep tracking of students' physical activity during out-of-school hours, such as after school and weekends, through regular communication with parents and students to prevent experimental errors caused by sudden changes in subjects' physical activity (e.g., participation in or withdrawal from sports clubs, etc.).

### Measure method

This study measured the participants' uncorrected distance visual acuity and kinetic visual acuity from four groups before the experimental intervention (before the first week) and after the experimental intervention (after the 16th week). In order to minimize the error of experimental data, the detection methods and process were strictly carried out in accordance with the standards, and the same person conducted all the measurement and recording of various data before and after the experiment.

#### Measurement of uncorrected distance visual acuity

Participants' uncorrected distance visual acuity test was completed by the full-time school doctor, using the light box of current “Standard Logarithmic Visual Acuity Chart” for testing, and the whole operation process followed the ophthalmic examination standards. The measurement result of the right eye was taken as the final value of the subjects' uncorrected distance visual acuity.

#### Measurement of kinetic visual acuity

Kinetic visual acuity was tested with XP.14-TD-J905, the kinetic visual acuity detector produced by Shanghai Hump Automation Technology Co., Ltd. The range of kinetic visual acuity was between 0.1 and 1.6, and the higher the value, the better the kinetic visual acuity. Before the eyesight test, the tester explained the operation method. The student sat in front of the detector and look through the measuring window. After the test began, students would see a Landolt ring in a bright yellow circular shape. The Landolt ring approached the subject from the front, moving from a 50 meters distance at a velocity of 30 km/h. The students were instructed to rapidly press the joystick when they identified the orientation of the Landolt ring (four directions: up, down, left, right). Only the correct measurement values were recorded. Then, each student was tested three times in a row, and the average value was taken as the final value of the student's kinetic visual acuity.

### Statistical analysis

Excel spreadsheet was used for data entry and collation, and SPSS25.0, statistical software, was used for data analysis. All data were expressed as mean ± standard deviation (M ± SD). Before the experiment, the data were tested for homogeneity, and the data obeyed normal distribution and had the same homogeneity of variance. Repeated-measures analysis of variance (ANOVA) and paired sample t-test were used to analyze the test results of participants' kinetic visual acuity and uncorrected distance visual acuity before and after the experiment. The significance size of main effects and interactions was evaluated using ηp2 to evaluate the significance size of main effects and interactions, in which ηp2 ≤ 0.06 was a small effect, 0.06 < ηp2 ≤ 0.14 was a medium effect, and ηp2 > 0.14 was a large effect. The significance level is α = 0.05.

## Results

### Intervention effect analysis of ciliary-muscle training with different frequencies on kinetic visual acuity

Table 3 presents the results of the repeated-measures ANOVA on participants' kinetic visual acuity. As seen in Table 3, the main effect of time was significant (F = 16.210, *p* < 0.05), which indicated that participants' kinetic visual acuity changed significantly with the passage of time. The interaction of time and group (shown as time × group) was significant (F = 3.405, *p* < 0.05), which indicated that under the interaction of time and group, different frequency of ciliary-muscle training in physical education had different effects on students' kinetic visual acuity.

**Table 3:** Results of repeated-measures ANOVA on participants' kinetic visual acuity.

**Elements**	**Value of F**	**Value of *P***	**η_p_^2^**
Time	16.210*	0.000	0.094
Group	1.727	0.164	0.032
Time × Group	3.405*	0.019	0.061

^*^indicates significant difference (p < 0.05).

**Figure 1:**
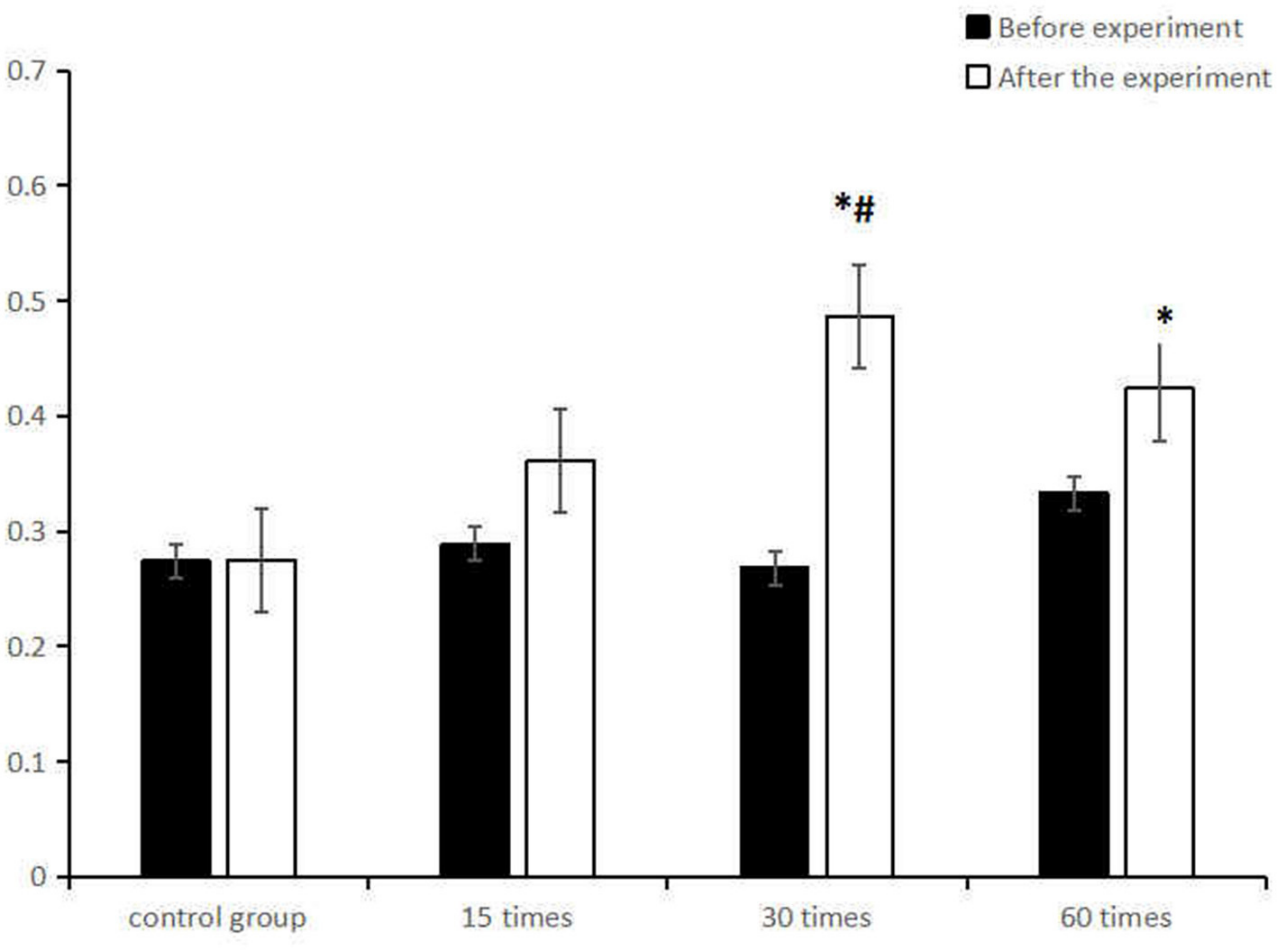
Comparison of the mean (M ± SD)kinetic visual acuity between the control group and the ciliary muscle training group with different frequencies before and after the experiment. *represents significant difference in intra-group comparison (*p* < 0.05), ^#^represents significant difference in comparison with the control group after the experiment (*p* < 0.05).

Further simple effect analysis (Table 4) found that at the level of baseline test, there was no statistically significant difference in kinetic visual acuity among the groups (F = 0.520, *p* > 0.05); At the post-test level, there was a significant difference in the kinetic visual acuity among the groups (F = 3. 701, *p* < 0.05). Multiple comparison shows there was a remarkable difference in the kinetic visual acuity between the control group and the 30-frequency group (*p* < 0.05). The paired sample T test of kinetic visual acuity before and after the experiment showed that the kinetic visual acuity of the students in both the 30 and the 60-frequency groups was significantly improved (*p* < 0.05), while that of the students in the 15-frequency group and the control group had no significant change (p>0.05) (Figure 1).

**Table 4:** Simple effect analysis and paired-sample *t*-test on subjects' kinetic acuity.

**Measure time**	**Groups**	**M ±SD**	**Value of F**	**Value of *P***	**η_p_^2^**
Pre-experiment	15-frequency group	0.289 ± 0.246	0.520	0.669	0.010
	30-frequency group	0.268 ± 0.214			
	60-frequency group	0.333 ± 0.304			
	Control group	0.274 ± 0.276			
Post-experiment	15-frequency group	0.361 ± 0.248	3.701	0.013	0.066
	30-frequency group	0.486 ± 0.327^ab^			
	60-frequency group	0.424 ± 0.324^a^			
	Control group	0.275 ± 0.200			

^a^means that compared with pre-experiment, p < 0.05.

^b^means significant difference in comparison with the control group after the experiment (p < 0.05).

### Intervention effect analysis of ciliary-muscle training with different frequencies on uncorrected distance visual acuity

Participants' uncorrected distance visual acuity in each group was analyzed by repeated measurement analysis of variance before and after the experiment, which reflected that the main effect of time was significant (F=14.076, *p* < 0.05), in other words, participants' uncorrected distance visual acuity changed significantly with the passage of time. Moreover, the interaction of time and group (shown as time × group) was significant (F=12.219, *p* < 0.05), which demonstrated that under the interaction of time and group, different frequencies of ciliary-muscle training in physical education had different effects on students' uncorrected distance visual acuity (Table 5).

**Table 5:** Repeated measures analysis of variance on participants' uncorrected distance visual acuity.

**Elements**	**Value of F**	**Value of *P***	**η_p_^2^**
Time	14.076*	0.000	0.083
Group	2.334	0.076	0.043
Time × Group	12.219*	0.000	0.190

*indicates significant difference (p < 0.05).

**Figure 2:**
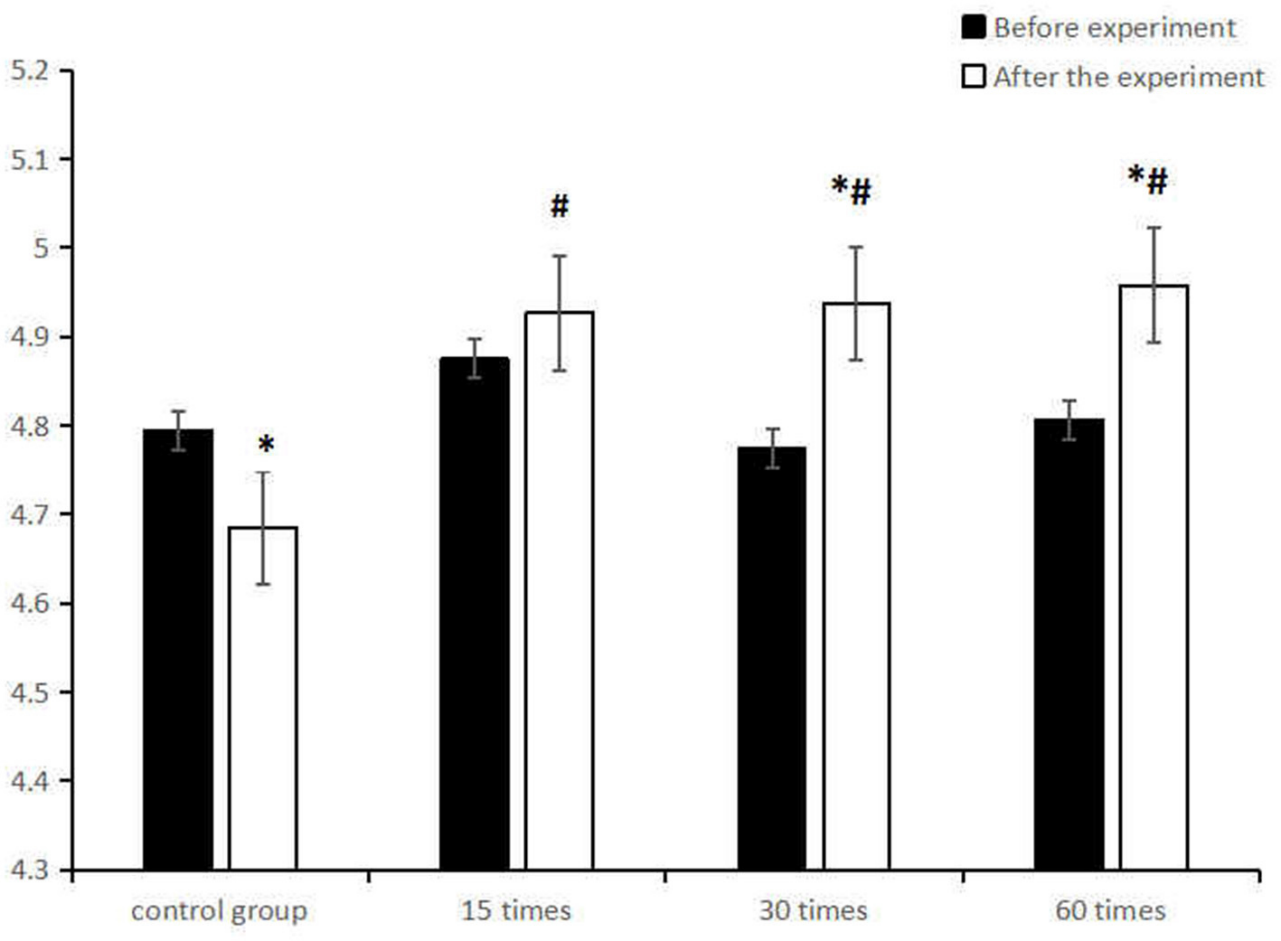
Comparison of the mean (M ± SD) uncorrected distance visual acuity between the control group and the ciliary muscle training group with different frequencies before and after the experiment. *represents significant difference in intra-group comparison (*p* < 0.05), ^#^represents significant difference in comparison with the control group after the experiment (*p* < 0.05).

Further simple effect analysis (Table 6) showed that at the level of baseline test, there was no statistically significant difference in uncorrected distance visual acuity among the groups (F = 0.776, *p* > 0.05); At the post-test level, a significant difference appeared in subjects' uncorrected distance visual acuity among the groups (F = 7. 095, *p* < 0.05), and there was a remarkable difference in the uncorrected distance visual acuity between the control group and the three experimental groups (*p* < 0.05). The paired sample *t* test of uncorrected distance visual acuity before and after the experiment found that students' uncorrected distance visual acuity in both the 30 and the 60-frequency groups was significantly improved (*p* < 0.05), while that of the students in the 15-frequency group had no significant change (p>0.05), but the control group displayed a significant decrease (*p* > 0.05) (Figure 2).

**Table 6:** Simple effect analysis and paired-sample *t*-test on subjects' uncorrected distance visual acuity.

**Measure time**	**Groups**	**M ±SD**	**Value of F**	**Value of *P***	**η_p_^2^**
Pre-experiment	15-frequency group	4.875 ± 0.334	0.776	0.509	0.015
	30-frequency group	4.775 ± 0.306			
	60-frequency group	4.807 ± 0.335			
	Control group	4.794 ± 0.302			
Post-experiment	15-frequency group	4.927 ± 0.311	7.095	0.000	0.120
	30-frequency group	4.937 ± 0.269^ab^			
	60-frequency group	4.958 ± 0.280^ab^			
	Control group	4.685 ± 0.277^ab^			

^a^means that compared with pre-experiment, p < 0.05.

^b^means significant difference in comparison with the control group after the experiment (p < 0.05).

## Discussion

### Development of 10–11-year-old children's visual health

More attention should be paid to the relationship between visual acuity and age in terms of prevention and cure for children's myopia, as the development of vision shows different characteristics in different age groups. Before the age of 6, it is the key development period of children's uncorrected distance visual acuity (25). Later, at the age of 10–14, their uncorrected distance visual acuity decreases significantly with age (26). It should be noted that myopia develops rapidly among children aged 7–12 years (27). A scholar investigated the current status of children's poor vision in a Chinese city and found that the rate of poor vision among children aged 9–12 was as high as 51.28% (28). This study showed that participants' overall visual health in the control group without any intervention displayed a downward trend, which may be related to the stage characteristics of visual development and the abnormal adjusting function of eyes. According to Kepler's near-work hypothesis (29), myopia was based on the continuous contraction of ciliary muscle, which created higher intraocular pressure during overloaded near work. Children's myopia took shape around school-age was mostly caused by ciliary-muscle dysfunction, coincidentally the peak period of myopic morbidity coincided with the peak period of individual's physical growth and development and also the individual's high-intensity academic learning (30, 31). In addition, external environmental factors such as near work, education intensity, long screen time, lack of physical activity and outdoor exercise were all recognized as risk factors for children's myopia. Particularly, children aged 10–11 are just in the period of rapid physical growth and development, and at the same time they step into the stage of increasing academic pressure, with more time for close work and less time for outdoor physical activities. In this situation, their ciliary muscle cannot be fully relaxed due to continuous contraction, consequently the range, flexibility and accuracy of ciliary-muscle regulation are reduced or its regulation ability will lose, and that's why children's visual acuity is prone to fluctuation. It is no doubt that at this stage, if we can provide helpful treatment strategies, the prevention and control of children's myopia can achieve better benefits.

### Effect of physical activity combined with ciliary-muscle training on children's visual health

The present study found that ciliary-muscle training in physical education class had a positive effect on improving children's visual health. As is known, the root of myopia is ciliary muscle spasm. Fortunately, special ciliary-muscle training can relieve this problem. In the past, some previous researchers were misled to believe that ciliary muscle cannot be changed by exercise, since structurally ciliary muscle belongs to smooth muscle (32). However, in recent years, it has been found that the muscle cells of ciliary muscle are different from the smooth muscle of other parts such as blood vessels, which are regularly arranged dense bands and dense bodies, similar to the Z band in striated muscle; moreover, in the ultrastructure, the number of mitochondria and endoplasmic reticulum in ciliary muscle cells is more abundant than that in ordinary smooth muscle (33). Due to the fact that ciliary muscle not only has the smooth muscle characteristics controlled by parasympathetic nerve, but also has many physiological structure, histochemistry and efferent nerve characteristics of striated muscle, increasing scholars convince that it can be regulated by special training.

Physical activity is the best medium for ciliary-muscle training in childhood. Studies have found that both open-skill sports and closed-skill sports can effectively improve students' kinetic visual acuity and uncorrected distance visual acuity after adding visual tasks (24). Besides, ball games with the nature of open-skill sports are accompanied by the alternation of close and distant vision in the process of practice, in which the ciliary muscle needs to be constantly adjusted to make the vision clear, which is conducive to the recovery of regulatory function (34). Admittedly, compared with open movement, closed movement requires less vision adjustment in the process of practice, but embedding the task of alternating near-far vision in closed movement, and increasing the frequency of eye use in closed movement can also improve the vision of myopic students (23).

According to the findings of previous studies (24), it is not difficult to see that different types of sports can show different effects on improving children's visual acuity, and this kind of differentiation is likely to be related with the frequency of ciliary-muscle training during exercise. In addition, some scholars (35) believed that in the process of physical activities, the adaptation of human eyes to changes in the external environment increased the frequency of ciliary muscle regulation, coupled with the strengthening of nerve regulation and the relaxation of psychological state, which may improve the spasm of ciliary muscle to a certain extent, thus having a positive impact on the level of visual health.

### Effects of ciliary-muscle training with different frequencies on children's kinetic visual acuity and uncorrected distance visual acuity

The results of this study showed that different frequencies of ciliary-muscle training had different effects on students' visual acuity. That was partly because of different amount of ciliary-muscle regulation triggered by different training intensity. In fact, the process of visual training is essentially the process of sports training, and the only main difference is that the training part concentrated on eyes. Although the eye muscle group belongs to the small muscle group, it can still support a suitable amount of exercise, so different amount of exercise done by the ciliary-muscle will create diverse stimulation, which is why in a certain period of intervention, different training frequencies have different effects on vision.

Another finding of this study was that 30 times of ciliary-muscle training in each physical education class had the best effect on improving students' kinetic visual acuity, and 30 times and 60 times of ciliary-muscle training had similar effects on improving students' the uncorrected distance visual acuity. In general, ciliary-muscle training with 30-frequency had better effects on the two kinds of vision than that of the 15 and the 60-frequency groups, which can be well explained. From a physiological point of view, eye muscles belong to small muscle groups, with poor tolerance, fatigue and other characteristics, frequency of 30 may be the appropriate intensity for ciliary-muscle training with physical activities as the carrier, which is more likely to produce the ideal effect on improving the adjustment ability of ciliary muscle.

Also, from the experimental point of view, ciliary-muscle training with frequency of 30 can be widely accepted in the experiment, and children had a high degree of execution and can complete the experimental task with high quality. Through the intervention, the ciliary muscle was not only fully relaxed, but also the sensitivity and accuracy of eye adjustment can be enhanced. In comparison, the 60-frequency training group was affected by the longer experimental time, resulting in a lower degree of participants' execution, in which children were unable to fully adjust and relax their eyes, and the quality of task completion was worse than that of the 30-frequency group. As for the 15-frequency group, 15 times of ciliary-muscle training can only make the strained and spasmodic ciliary muscle experience a simple warm-up effect. After 15 times of training, intervention on the ciliary muscle immediately stopped, but the muscle just reached the state of preparation, so the relaxation of ciliary muscles failed to continue, resulting in insufficient stimulation intensity. Therefore, 15-frequency training failed to reach the threshold of cillary muscle training. Based on the explanation above, it is a more reasonable and scientific choice to take 30 times in each physical class as the benchmark threshold for ciliary-muscle training combined with physical activities. The reason for the visual acuity loss in the control group may be that Chinese students have fewer opportunities to participate in extracurricular physical activities, and the ciliary muscle conditioning in daily school physical activities cannot counteract the negative effects of students due to close eye use and excessive time spent on visual screens.

At present, the traditional teaching mode of physical education at school has limited effect on improving children's visual acuity. Being aware of this undesirable situation, this study asserts that it is necessary to combine ciliary-muscle training with regular physical activities to form a new physical education teaching mode that not only enables children to master sports skills, but also works in children's myopia prevention and control. By this way, it will motivate children's active participation in the new mode, and help them cultivate a good habit of self-regulation to improve myopia.

Although great effort was made, the present study was far from satisfactory. Only 10–11-year-old children were selected for the experiment, but no comparative study of multi-population, larger sample and different methods was carried out. Due to these limitations, in terms of research content, it is recommended that future studies further explore the effects of different intervention frequencies on the visual acuity levels of children at various ages, even refining to different genders and visual acuity levels, and also expanding to the relationship between different intervention hourly values and intensities and the visual acuity levels of children and adolescents at various school levels. All of the aspects are worth exploring experimentally. In terms of related index measurements, future studies can perform ciliary muscle paralysis on the measurement subjects to obtain more accurate data.

## Conclusions

On the basis of the experiment data analysis, this study revealed that physical activity combined with extra ciliary-muscle training has a positive effect on improving children's visual health. At the same time, different frequencies of ciliary-muscle training produces different effects on children's visual acuity. In particular, the 30-frequency ciliary-muscle training is the optimal choice to improve children's kinetic visual acuity and uncorrected distance visual acuity in each physical education class.

## Data availability statement

The data that support the findings of this study are available on request from the first author. The data are not publicly available because they contain information that could compromise the privacy of research participants. Requests to access the datasets should be directed to RY, yrb@suda.edu.cn.

## Ethics statement

The studies involving human participants were reviewed and approved by the Ethics Committee of Soochow University. Written informed consent to participate in this study was provided by the participants' legal guardian/next of kin.

## Author contributions

Conceptualization and validation: RY and JX. Methodology and writing—original draft preparation: RY. Formal analysis and data curation: SZ and MZ. Investigation and visualization: HW. Resources: GC. Writing—review and editing: JX. Supervision: RY and JX. Project administration and funding acquisition: GC. All authors have read and agreed to the published version of the manuscript.

## Funding

This research was supported by the National Social Science Fund of China (No. 19BTY078).

## Conflict of interest

The authors declare that the research was conducted in the absence of any commercial or financial relationships that could be construed as a potential conflict of interest.

## Publisher's note

All claims expressed in this article are solely those of the authors and do not necessarily represent those of their affiliated organizations, or those of the publisher, the editors and the reviewers. Any product that may be evaluated in this article, or claim that may be made by its manufacturer, is not guaranteed or endorsed by the publisher.
